# A Pilot Study of the Role of Semaphorin 4A (sema4A) and 3C (sema3C) in Non-Muscle-Invasive Bladder Cancer (NMIBC)

**DOI:** 10.3390/biomedicines12102407

**Published:** 2024-10-21

**Authors:** Piotr Purpurowicz, Tomasz W. Kaminski, Władysław Kordan, Anna Korzekwa, Zbigniew Purpurowicz, Zbigniew Jabłonowski

**Affiliations:** 1 Department of Urology and Urological Oncology, Municipal Hospital in Olsztyn, 10-045 Olsztyn, Poland; purp8@wp.pl; 2 Pittsburgh Heart, Lung and Blood Vascular Medicine Institute, University of Pittsburgh, Pittsburgh, PA 15260, USA; tkaminski@versiti.org; 3 Thrombosis and Hemostasis Program, VERSITI Blood Research Institute, Milwaukee, WI 53226, USA; 4 Department of Animal Biochemistry and Biotechnology, Faculty of Animal Bioengineering, University of Warmia and Mazury in Olsztyn, 10-719 Olsztyn, Poland; wladyslaw.kordan@uwm.edu.pl; 5 Research Group of Biodiversity Protection, Institute of Animal Reproduction and Food Research of Polish Academy of Sciences in Olsztyn, 10-748 Olsztyn, Poland; a.korzekwa@pan.olsztyn.pl; 6 1st Department of Urology, Medical University of Łódź, 90-549 Łódź, Poland; zbigniew.jablonowski@umed.lodz.pl

**Keywords:** semaphorin, sema4A, sema3C, bladder cancer, non-muscle-invasive bladder cancer, biomarker

## Abstract

**Background/Objectives:** Bladder cancer is a very important issue in contemporary urology. The aim of this pilot study was to assess for the first time the clinical utility of semaphorin 3C (sema3C) and 4A (sema4A) in patients with non-muscle-invasive bladder cancer (NMIBC). **Methods:** The experiment involved 15 patients with NMIBC and 5 patients without malignancies as the control group. Plasma and urinary concentrations of sema3C and sema4A were assessed by using an enzyme-linked immunosorbent assay (ELISA). Urinary sema4A concentration was below the detection level. **Results:** There was no statistically significant difference between patients and controls in terms of plasma sema4A and sema3C or urinary sema3C concentrations (*p* > 0.05). There was a significantly higher sema3C plasma concentration in patients with low-grade tumors (*p* = 0.0132) and an upward trend in sema4A plasma concentration for the subjects with Ta-stage tumors. Urinary sema3C concentration positively correlated with tumor size (R = 0.57, *p* = 0.03). Plasma sema3C concentration correlated negatively with tumor grade (R = −0.62, *p* = 0.01). **Conclusions:** Urinary sema4A concentration, which is below the detection threshold, is unlikely to be useful as a marker of NMIBC. Plasma sema4A concentration and sema3C concentration in plasma and urine cannot be used as stand-alone markers of NMIBC at this point. The plasma concentration of sema3C can potentially be considered in the future as a marker for tumors of lower grades. Plasma sema4A concentration could potentially be considered in the future as a marker for tumors of earlier stages. All of these observations are preliminary, so they have to be assessed in larger cohorts to make reliable recommendations. Nevertheless, our study lays the groundwork for further research to develop potential tests that could be used in daily practice to monitor and predict the course of cancer.

## 1. Introduction

Bladder cancer is one of the most significant neoplasms in contemporary urology. It is the most frequent cancer in the urinary tract and is notably more common in men than in women [1,2]. Its classical symptoms are hematuria and painful urination [3]. The recognized risk factors are tobacco smoking and occupational exposure [4]. Surprisingly, there are reports on the protective role of cardiovascular diseases [4]. The most important examination, the gold standard, in the diagnostic process is cystoscopy with histopathological examination of samples from the suspicious lesions [3]. Non-muscle-invasive bladder cancer (NMIBC) is a particular type of bladder cancer which is a tumor that is limited to the mucosa and submucosa and does not penetrate through the muscle layer. This type is most common among patients, and more than 70% are diagnosed at this stage [2,4]. The treatment of bladder cancer depends the grading and staging of the tumor, as well as the patient’s general condition and accompanying diseases. It comprises the transurethral resection of the bladder tumor (TURBT), cystectomy, BCG therapy, chemotherapy, or radiotherapy [3,5]. Unfortunately, the 5-year survival rate in localized bladder cancer is only 30%, which highlights the need for early detection and treatment [6].

Semaphorins are a broad group of extracellular proteins which are engaged in various biological process. The common feature of all family members is the sema domain. They are involved in neural network and cardiovascular system development, immunity, bone homeostasis, and tumorigenesis [7].

Sema3C is a member of class III semaphorins which are secretory proteins in humans. Sema3C’s role has been proved in immunological processes and nervous and cardiovascular system development [8]. In tumorigenesis, sema3C seems to have a multifaced nature. There are reports on sema3C promotion in cases of prostate and gastric cancer, as well as glioma [7], often associated with unfavorable prognosis [9]. On the other hand, there is information about its suppressive influence, especially due to the inhibition of tumor-associated angiogenesis [7]. So far, sema3C has never been evaluated in bladder cancer.

Sema4A is a member of class IV semaphorins which are transmembrane proteins. It is involved in inflammatory processes, nervous system development, and angiogenesis [8]. In particular, its role in the latter is highlighted since it is a vital anti-angiogenic agent which inhibits the development of malignant tumors, whereas sema4A downregulation leads to neoplasm progression [10]. Similar to sema3C, sema4A has never been studied in bladder cancer.

The aim of this study was to assess the potential role of sema4A and sema3C in NMIBC. We have already explored the role of sema6D in bladder cancer [11] and proved it could be a potential urinary marker of this neoplasm. Encouraged by our previous results, we would like to determine the potential role of the other two semaphorins. Moreover, the design of this study makes it possible to easily translate the obtained observations into daily clinical practice and potentially apply the findings in cancer patients.

## 2. Materials and Methods

The study group involved 15 patients (10 men, 5 women) with an average age of 63.73 years. The following inclusion criteria were set: age over 18 years, and NMIBC diagnosed for the first time, confirmed by histopathological examination. The exclusion criteria were as follows: age under 18 years, pregnancy or breast-feeding, past history of malignancy (including NMIBC), muscle-invasive bladder cancer (MIBC), and immunosuppressive drug intake.

Following the administration of general anesthesia or a subarachnoid spinal block, all patients underwent a transurethral resection of a bladder tumor (TURBT). This procedure involved obtaining tumor tissue for a histopathological examination to verify the presence of bladder cancer and rule out patients with MIBC.

The control group of 5 patients (3 men, 2 women) without NMIBC (and any other malignancy) was matched with the study group in terms of gender and geographical location. The criteria for inclusion were individuals aged over 18 years who did not have NMIBC or MIBC as confirmed by cystoscopy, had no prior history of other malignancies, and were not taking immunosuppressants. In the control group, the absence of bladder tumors was verified during non-cancer-related procedures such as transurethral resection of the prostate (TURP), through ureterorenoscopic lithotripsy, or through a negative cystoscopy in patients suspected of having a bladder tumor.

Blood and urine samples were collected at the beginning of the study both from the study and control group. Fasting blood samples were collected using vacuum tubes. They were centrifugated for 10 min at 2000× *g*. Urine samples were collected as first morning specimens from mid-stream and were centrifugated for 10 min at 2000× *g*. The obtained plasma and urine were stored at −80 °C until the analysis. Sema3C and sema4A concentrations were measured by using an enzyme-linked immunosorbent assay (ELISA) with commercially available kits according to the manufacturer’s protocol. Each run was performed in duplicate, and the average was considered a single sample. Sema3C concentration was measured by the sema3C kit provided by Aviva Systems (OKD02877, San Diego, CA, USA). The detection range was estimated to be 78–5000 pg/mL, and the sensitivity was 6.7 pg/mL. Sema4A concentration was measured by the sema4A kit provided by Cloud Clone (SEL921Hu, Wuhan, China), the range was estimated to be 31.2–2000 pg/mL, and the sensitivity was 12.7 pg/mL. The absorbance was read at 450 nm. All experiments were conducted by the same investigator in the same laboratory setting.

The collected data underwent Shapiro–Wilk normality testing, and all of the data with Gaussian distribution are presented as the mean ± SE. The correlation results have been generated using the Pearson Correlation Coefficient or Spearman’s Rank Test, as appropriate. Values with *p* < 0.05 were considered statistically significant. The data were analyzed using GraphPad Prism 8.0 Software (La Jolla, CA, USA).

The research was conducted following the guidelines of the Helsinki Declaration [12] and was approved by the Bioethics Committee of the Medical University of Lodz, Poland (no RNN/31/23/KE).

## 3. Results

For this pilot study, we recruited 15 patients and 5 volunteers as the control group, with their baseline parameters listed below (Table 1).

There was no statistically significant difference between the patients and controls in terms of sex and kidney function; however, there was a weak difference in terms of the mean age.

The participants’ plasma and urine were tested twice by using an ELISA for sema3C and sema4A. Urinary sema4A concentration was below the detection level so it was not further analyzed.

**Figure 1:**
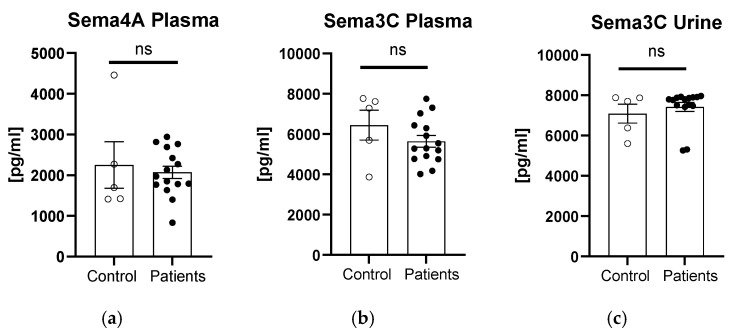
Concentration of semaphorins in patients compared to controls: sema4A in plasma (**a**), and sema3C in plasma (**b**) or urine (**c**). ns, non-significant difference.

There was no statistically significant difference between the patients and controls in terms of plasma sema4A and sema3C or urinary sema3C concentration (*p* > 0.05, Figure 1a–c).

**Figure 2:**
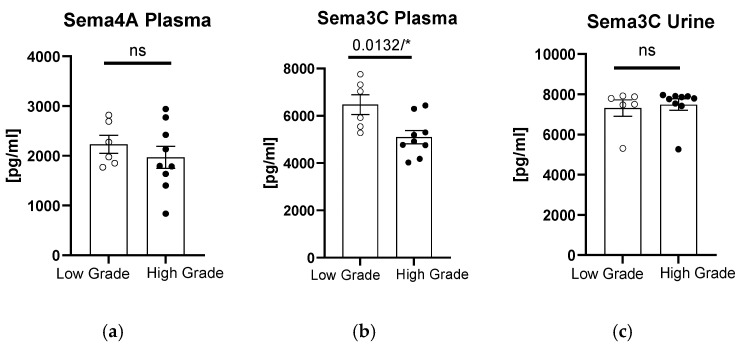
Concentration of semaphorins in patients compared to controls after division according to tumor grading: sema4A in plasma (**a**), and sema3C in plasma (**b**) or urine (**c**). * means statistically significant difference with *p* < 0.05; ns, non-significant difference.

When we divided the patients according to the grade of bladder tumor, we observed significantly higher sema3C plasma concentrations in patients with low-grade tumors (*p* = 0.0132, Figure 2b). In terms of plasma sema4A or urinary sema3C concentrations, there were no significant differences between the patients and controls (*p* > 0.05, Figure 2a,c).

**Figure 3:**
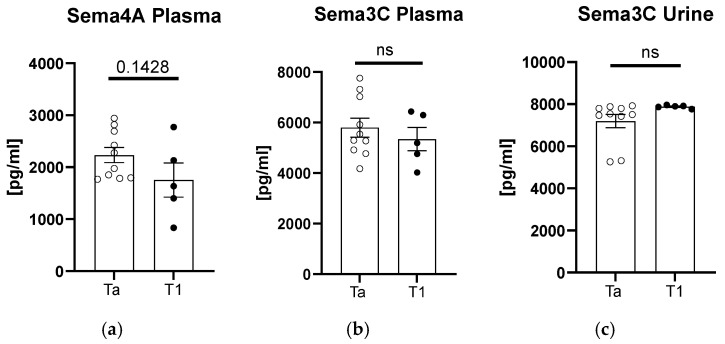
Concentration of semaphorins in patients compared to controls after division according to tumor staging: sema4A in plasma (**a**), and sema3C in plasma (**b**) or urine (**c**). ns, non-significant difference.

After the division of patients according to tumor staging, we found an upward trend in the sema4A plasma concentration for the subjects with Ta-stage tumor (Figure 3a). In terms of plasma and urinary sema3C concentrations, there were no significant differences between the patients and controls (*p* > 0.05, Figure 3b,c).

**Figure 4:**
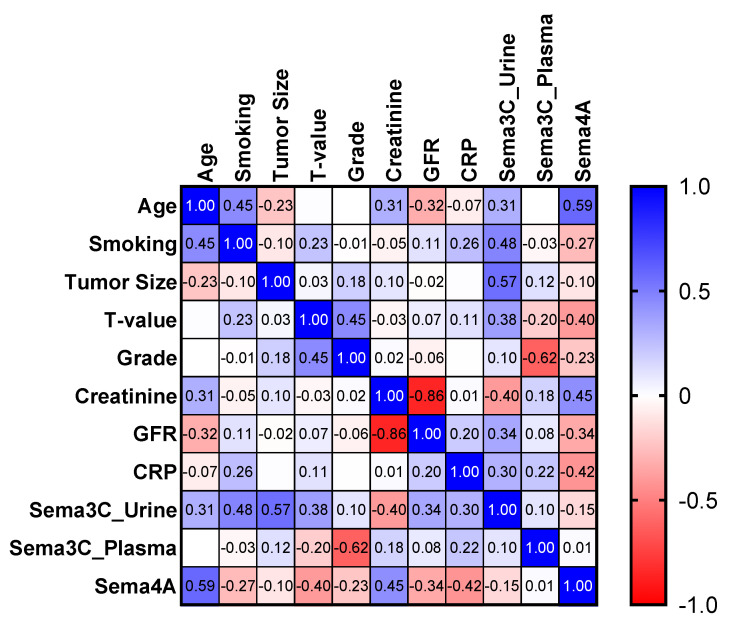
Diagram of Spearman’s rank correlations.

We also searched for correlations between semaphorin concentrations and available clinical or laboratory parameters (Figure 4). Urinary sema3C concentration positively correlated with tumor size (R = 0.57, *p* = 0.03). There was also an upward trend for urinary sema3C and smoking (R = 0.48, *p* = 0.07). Plasma sema3C concentration correlated negatively with tumor grade (R = −0.62, *p* = 0.01). Plasma sema4A concentration positively correlated with the age of patients (R = 0.59, *p* = 0.02).

We also attempted to search for predictors of sema3C or sema4A concentrations; however, we did not find any significant factor (Table 2).

## 4. Discussion

In our previous experiment, we proved the potential utility of urinary sema6D as it was significantly higher in patients with NMIBC compared to controls [11]. This study, on the other hand, is a preliminary investigation of the potential role of sema3C and sema4A in bladder cancer. However, we did not observe significant differences in semaphorin concentrations between the patients and controls, although we obtained some interesting findings that could be studied more deeply in the future to determine their clinical utility. The only non-invasive test that has gained its actual place in urological practice is urine cytology. However, it is characterized by high specificity, and its sensitivity is low [13], so better tests are necessary.

To the best of our knowledge, sema3C and sema4A have never been studied among patients with bladder cancer, so we are the first to report on this matter.

Among all urological cancers, both sema3C and 4A have been studied in prostate cancer. Sema4A was documented to promote prostate cancer invasion. It was overexpressed in cancer cells and even correlated with Gleason score [14]. Sema3C also seems to be involved in the progression of prostate cancer via the mutation of the transcription factor FOXA1 and even contributes to its resistance to therapy [15,16]. Sema3C could be considered a future therapeutic target [16].

In our study, urinary sema4A concentration was below the detection threshold; therefore, it is unlikely to become useful as a marker of NMIBC. The lack of in-depth information regarding kidney metabolism of sema4A, leading to its urinary secretion, makes it difficult to interpret this observation at this point.

Plasma sema4A concentration and sema3C concentration in plasma and urine cannot be used as stand-alone markers of NMIBC at this time due to no statistically significant differences between the patients and controls.

Interesting observations were made about sema3C and sema4A correlations with clinical parameters; however, considering that neither of them was significantly different between the patients and controls, these findings must be approached with caution and studied in larger groups. Nevertheless, taking into account the nature of bladder cancer and its tendency to reoccur, measures must be taken to lower the number of invasive procedures related to assessing the diagnosis and further grading and staging of the tumor [2]. So far, there have been several non-invasive tools proposed; however, they have not been very popular in the clinical setting. They are mainly based on the detection of gene mutations, chromosome abnormalities, or mRNA. Only a few comprise the detection of specific proteins [2], which is easier to perform and thus cheaper to translate into clinical practice.

Considering that the plasma concentration of sema3C was significantly higher in patients with low-grade tumors and that it correlated negatively with this grade, sema3C can potentially be considered in the future as a marker for tumors of lower grades. It would have to be tested in larger cohorts; however, it is a potentially clinically useful observation, since lower sema3C concentrations could predict high-grade tumors. Interestingly, urinary sema3C concentration positively correlated at the same time with tumor size, so it seems to become higher in subjects with bigger lesions. It could be potentially explained in such a way that low-grade tumors grow slower, eventually reaching greater volumes which translate to higher amounts of released sema3C.

Plasma sema4A concentration could potentially be considered in the future as a marker for tumors of earlier stages. Similar to sema3C, more in-depth studies are required to establish its clinical application, but its lower concentrations could perhaps indicate higher NMIBC stages, and the other way round—higher concentrations could be predictive of earlier stages and better prognoses. It has an essential clinical application with prognostic purposes, since the 5-year relative survival rates for bladder cancer are 97% in cases of carcinoma in situ and 71% for localized bladder cancer [17].

Our study lays the groundwork for further research to develop potential tests that could be used in daily practice to monitor and predict the course of cancer.

The main limitation of this study is the relatively small number of participants enrolled, especially in the control group. The preliminary nature of this study and the strict enrolling criteria are the main reasons for the occurrence of this limitation. Although the results lack of robust significance, the presented data deliver novel insights into the possible role of sema4A as a biomarker for early stages of tumors. Future extended studies could further explore whether distinct genomic subtypes of bladder cancer exhibit differential responses to semaphorin signaling. This could be particularly relevant for therapeutic targeting or understanding resistance mechanisms.

## 5. Conclusions

Urinary sema4A concentration, which is below the detection threshold, is unlikely to be useful as a marker of NMIBC. Plasma sema4A concentration and sema3C concentration in plasma and urine cannot be used as stand-alone markers of NMIBC at this point. The plasma concentration of sema3C can potentially be considered in the future as a marker for tumors of lower grades. Plasma sema4A concentration could potentially be considered in the future as a marker for tumors of earlier stages. All of these observations are preliminary, so they have to be assessed in larger cohorts to make reliable recommendations.

## Figures and Tables

**Table 1:** Basic information about the participants.

Parameter	Controls (*n* = 5)	Patients (*n* = 15)
Sex (M/F)	3/2	10/5
Age (years)	53.4 ± 4.97	63.73 ± 2.1 (0.0375 *)
Creatinine (mg/dL)	0.83 ± 0.05	0.88 ± 0.07
eGFR	94 ± 5.98	92.08 ± 6.95

* means significant difference with *p* < 0.05.

**Table 2:** Predictors of sema3C and sema4A.

Parameter Estimates	Variable	Estimate	Standard Error	95% CI (Asymptotic)	|t|	*p* Value	*p* Value Summary
β0	Intercept	18.06	36.90	−99.37 to 135.5	0.4893	0.6582	ns
β1	Tumor size	−0.2445	0.6257	−2.236 to 1.747	0.3907	0.7221	ns
β2	T	−0.2983	7.251	−23.37 to 22.78	0.04114	0.9698	ns
β3	Grade	−1.425	5.851	−20.04 to 17.19	0.2435	0.8233	ns
β4	Creatinine	17.95	16.65	−35.04 to 70.93	1.078	0.3600	ns
β5	GFR	0.006159	0.1360	−0.4268 to 0.4391	0.04527	0.9667	ns
β6	CRP	0.2572	0.7889	−2.253 to 2.768	0.3260	0.7658	ns
β9	Sema3C	0.005549	0.003064	−0.004202 to 0.01530	1.811	0.1678	ns
β10	Sema3C	−0.001989	0.001910	−0.008068 to 0.004091	1.041	0.3744	ns
β11	Sema4A	5.667	4.139	−7.506 to 18.84	1.369	0.2645	ns

ns, non-significant difference.

## Data Availability

The original contributions presented in the study are included in the article, further inquiries can be directed to the corresponding author.
